# Anti-proliferative, pro-apoptotic and anti-invasive effect of EC/EV system in human osteosarcoma

**DOI:** 10.18632/oncotarget.17089

**Published:** 2017-04-13

**Authors:** Francesca Punzo, Chiara Tortora, Daniela Di Pinto, Iolanda Manzo, Giulia Bellini, Fiorina Casale, Francesca Rossi

**Affiliations:** ^1^ Department of Women, Child and General and Specialist Surgery, Second University of Naples, 80138 Naples, Italy; ^2^ Department of Experimental Medicine, Division of Pharmacology “Leonardo Donatelli”, The Second University of Naples, 80138 Naples, Italy

**Keywords:** EC/EV system, osteosarcoma, OS cell lines, RTX, JWH-133

## Abstract

Osteosarcoma is the most common and aggressive bone tumor in children. The Endocannabinoid/Endovanilloid system has been proposed as anticancer target in tumor of different origins. This system is composed of two receptors (CB1 and CB2), the Transient Potential Vanilloid 1 (TRPV1) channel and their ligands and enzymes. CB1 is expressed mainly in central nervous system while CB2 predominantly on immune and peripheral cells. We investigated the effects of JWH-133 (CB2 agonist) and RTX (TRPV1 agonist) in six human Osteosarcoma cell lines: MG-63, U-2OS, MNNG/HOS, Saos-2, KHOS/NP, Hs888Lu, by Apoptosis and Migration-Assay. We also compared the effects of these compounds on Caspase-3, AKT, MMP-2 and Notch-1 regulation by Q-PCR and Western Blotting.

We observed an anti-proliferative, pro-apoptotic, anti-invasive effect. Our results show that both CB2 stimulation and TRPV1 activation, in different Osteosarcoma cell lines, can act on the same pathways to obtain the same effect, indicating the Endocannabinoid/Endovanilloid system as a new therapeutic target in Osteosarcoma.

## INTRODUCTION

Osteosarcoma (OS), the most common primary malignant tumor of bone, affects predominantly children and adolescents, exhibiting high invasion and metastasis rate [1, 2]. Due to a high frequency of systemic spread at the early phase and the strong chemotherapy resistance, the five-year survival rate remains at only 20%. Available treatments results in significant morbidity (cardiac toxicity, infertility, renal dysfunction) and chemo-resistance [3–6]. Moreover OS patients present a decrease in bone mass density (BMD) during chemotherapy, and in long-term survivors osteoporosis and fractures are frequently present [7, 8]. Therefore, novel therapeutic approaches are needed to treat OS more efficiently and improve patient's life quality.

The cell of origin of OS, is still far from clear. During the last 10 years mounting evidence has placed mesenchimal stem cells (MSCs) and/or their immediate lineage progenitors as the most likely cell-of-origin for many types of sarcomas including OS [9–11].

The Endocannabinoid/Endovanilloid (EC/EV) system has been proposed as an anticancer target by several studies [12–26]. This system consists of two G protein-coupled receptors, named CB1 and CB2, the transient receptor potential vanilloid 1 (TRPV1) channel, a non selective cation channel, their endogenous ligands and enzymes for their synthesis and inactivation. CB1 receptors are expressed at high levels in Central Nervous System, while the CB2 receptors are primarily expressed on immune and peripheral cells. TRPV1 has been described as an additional receptor target for several cannabinoids [27–29]. Evidences on the cross-talk between CB2 receptors and TRPV1 channels have been demonstrated [30–34]. Cannabinoids have been reported as potential antitumor compounds based on their ability to reduce inflammation, migration, cell proliferation and cell survival at a low dosage. Specifically, these effects are mediated mainly by CB2 receptors [15, 19, 21, 22, 35, 36].

However, we cannot exclude the effects that these compounds may induce, activating TRPV1 receptor channel. When TRPV1 channel proteins are activated, they mainly cause the calcium ion influx. Intracellular calcium overload mediate cell apoptosis through different mechanism interfering with cell energy production and metabolism, therefore also drugs acting on the TRPV receptors can act as potential target to reduce cell proliferation and survival in cancer [37–39].

Recent studies described the role of EV/EC system in remodelling, differentiation, survival and activity of bone cells. In particular, we have demonstrated a functional cross-talk between CB2 and TRPV1 in human osteoclasts. TRPV1 stimulation, exerts pro-osteoclastogenic effects, whereas CB2 stimulation promotes anti-osteoclastogenic events, suggesting these receptors as new pharmacological target for the treatment of osteoporosis [32–34]. Moreover we have also demonstrated the presence of these receptors on human osteoblasts and MSCs and their role in migration, viability and cytokine release [31, 40].

Based on these evidences, we investigated for the first time the expression of CB2 and TRPV1 receptors and the effects of JWH-133 and RTX, (agonists of the EC/EV system) at different concentrations, in six OS cell lines (Saos-2, MG-63, MNNG/HOS, KHOS/NP, Hs888Lu and U-2 OS), analyzing the response to these compounds on cell survival, invasion and migration capacity. We observed an anti-proliferative, pro-apoptotic and anti-invasive effect induced by EC/EV compounds in the studied OS cell lineages.

## RESULTS

### OS cell lines express EC/EV system

**Figure 1:**
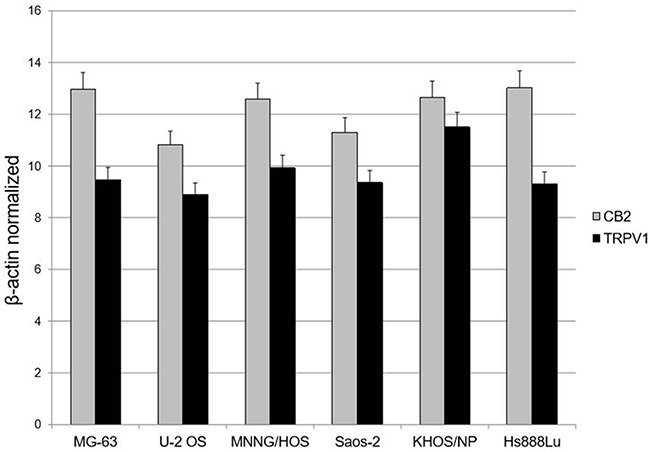
EC/EV receptors’ expression in OS cell lines OS cell lines express the Cannabinoid receptor type 2 (CB2) and the Transient Receptor Potential Vanilloid type-1 (TRPV1). Data have been revealed by Q-PCR, starting from 1000 ng of total mRNA for the RT reaction. Results were normalized for the housekeeping gene β-actin and were showed as mean ± SD of three independent experiments.

We performed a Real Time PCR to evaluate the expression of EC/EV system in untreated MG-63, U-2 OS, MNNG/HOS, Saos-2, KHOS/NP and Hs888Lu prior to the treatments, to be sure the receptors we were going to stimulate, were present. The result demonstrated the presence of mature mRNA for CB2 and TRPV1 receptors (Figure 1).

### Effect of EC/EV on OS cell lines viability and apoptosis

#### Annexin, Count and Viability

**Figure 2:**
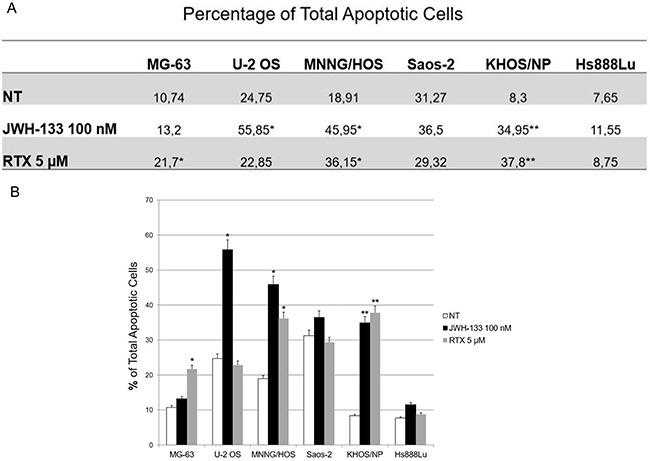
Effects of JWH-133 and RTX on apoptosis in OS cell lines **(A)** Mean percentage of total apoptosis, of three independent experiment, induced by JWH-133 [100 nM] and RTX [5 μM], after 24h of exposure. The graph **(B)** represents the mean percentage ± SD of total apoptosis, of three independent experiment, induced by JWH-133 [100 nM] and RTX [5 μM], after 24h of exposure. A t-test has been used to evaluate statistical differences among the groups. * indicates p ≤ 0.05 compared to the untreated control (NT), ** indicates p ≤ 0.01 compared to the untreated control (NT).

RTX and JWH-133 showed an effect on apoptosis in OS cell lines (Figure 2). JWH-133 100nM increased apoptosis in a significant manner, compared to the non-treated cell line (Figure 2A and Supplementary Figure 1). Whereas increasing concentrations of JWH-133 resulted in an increase in cell survival and decrease in apoptosis in all cell lines evaluated (Data not shown). RTX induces apoptosis (on average more than 50%) in MG63, KHOS/NP, MNNG/HOS, at the concentration of 5 μM (Figure 2B) while at 7 μM we could not observe any effect (Data not shown). For this reason, we proceeded treating OS cells only with RTX and JWH-133 at these concentrations (5 μM and 100nM respectively).

#### Caspase-3 expression levels

**Figure 3:**
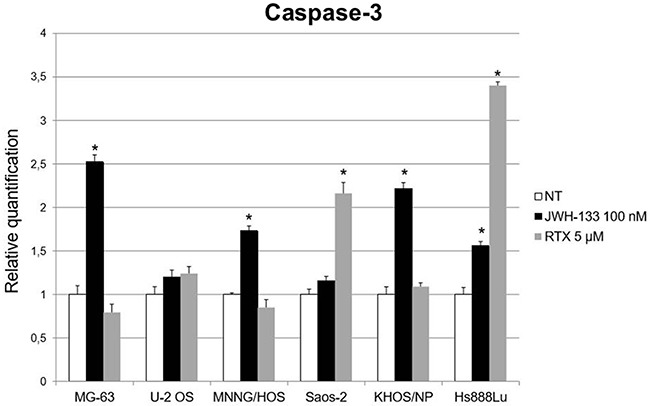
Effects on Caspase-3 mRNA expression levels of JWH-133 and RTX treatments Caspase-3 mRNA expression levels in OS cell lines were determined by Q-PCR after JWH-133 [100 nM] and RTX [5 μM] treatments. Results were normalized for the housekeeping gene β-actin and were showed as mean ± SD of three independent experiments. A t-test has been used to evaluate statistical differences in Caspase-3 expression among groups. * indicates p ≤ 0.05 compared to the untreated control (NT).

To evaluate the possible molecular mechanism through which EC/EV drugs act on apoptosis, we analyzed the expression levels of Caspase-3 after RTX [5 μM] or JWH-133 [100 nM] treatment. RTX induced a significant increase of Caspase-3 expression in Saos-2 and Hs888Lu cell lines as compared to respective control, while JWH-133 treatment resulted in a significant increase in Caspase-3 expression in MG-63, KHOS/NP, MNNG/HOS and also in Hs888Lu. In U-2 OS the Caspase-3 levels were also increased but the difference was not statistically significant (Figure 3).

**Figure 4:**
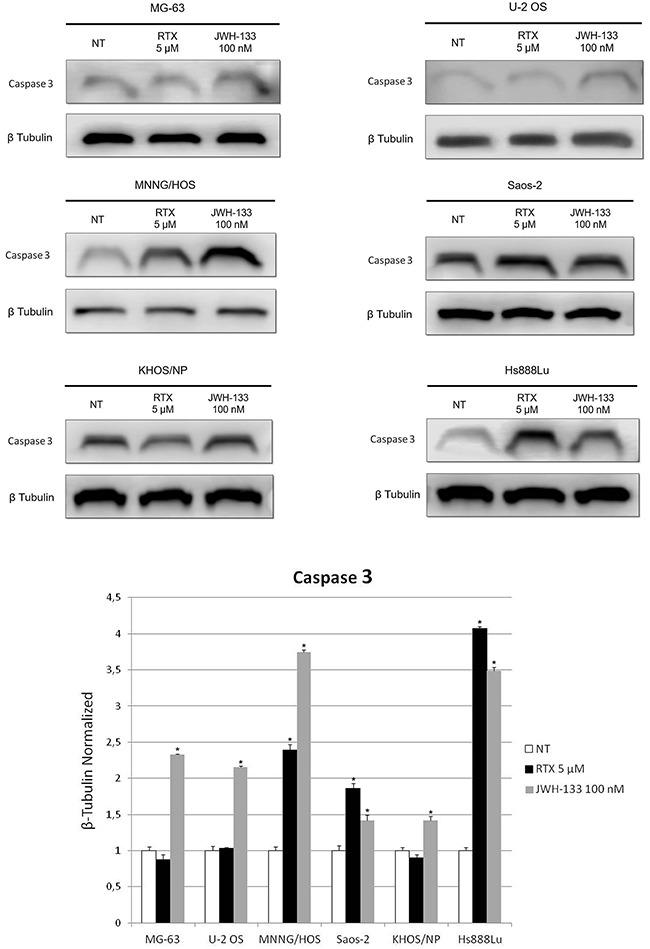
Effects of RTX and JWH-133 on Caspase 3 protein expression levels Caspase 3 protein expression levels in OS cell lines, determined by Western Blot, starting from 15 μg of total lysates after JWH-133 [100 nM] and RTX [5μM] treatments. The results were normalized for the housekeeping protein β-tubulin. The graph represents the mean and S.D. from two experiments. A t-test has been used to evaluate the statistical differences in protein expression levels. * indicates p ≤ 0.05 compared to the untreated control (NT).

Western Blotting confirmed the results obtained with Real Time PCR (Figure 4).

#### Akt protein expression level

**Figure 5:**
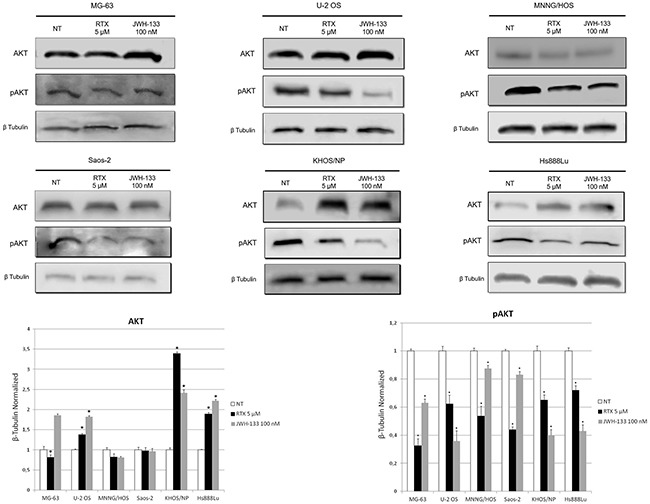
Effects of RTX and JWH-133 on AKT expression levels Total Akt and pAkt protein expression levels in OS cell lines, were determined by Western Blot, starting from 15 μg of total lysates after JWH-133 [100 nM] and RTX [5μM] treatments. The results were normalized for the housekeeping protein β-tubulin. The graph represents the mean and S.D. from two experiments. A t-test has been used to evaluate the statistical differences in protein expression levels. * indicates p ≤ 0.05 compared to the untreated control (NT).

We then investigated Akt expression levels. This pathway promotes survival and growth in response to extracellular signals. Activated Akt mediates downstream responses, including cell survival, growth, proliferation, cell migration and angiogenesis, by phosphorylating a range of intracellular proteins, so we investigated Akt and pAkt protein levels by western blot in OS cell lines total lysates. EC/EV compounds were added alone at concentrations of RTX [5 μM] and JWH-133 [100 nM]. Both drugs induced a reduction of pAKT expression level, but in MG-63, MNNG/HOS and Saos-2 the strongest effect was achieved with RTX, while in U2-OS, KHOS/NP and Hs888Lu the biggest reduction was achieved using JWH-133. Total Akt expression levels unchanged or increased after treatments, compared to the untreated sample (Figure 5).

### Effect of EC/EV on OS cells migration capacity

#### Scratch assay

**Figure 6:**
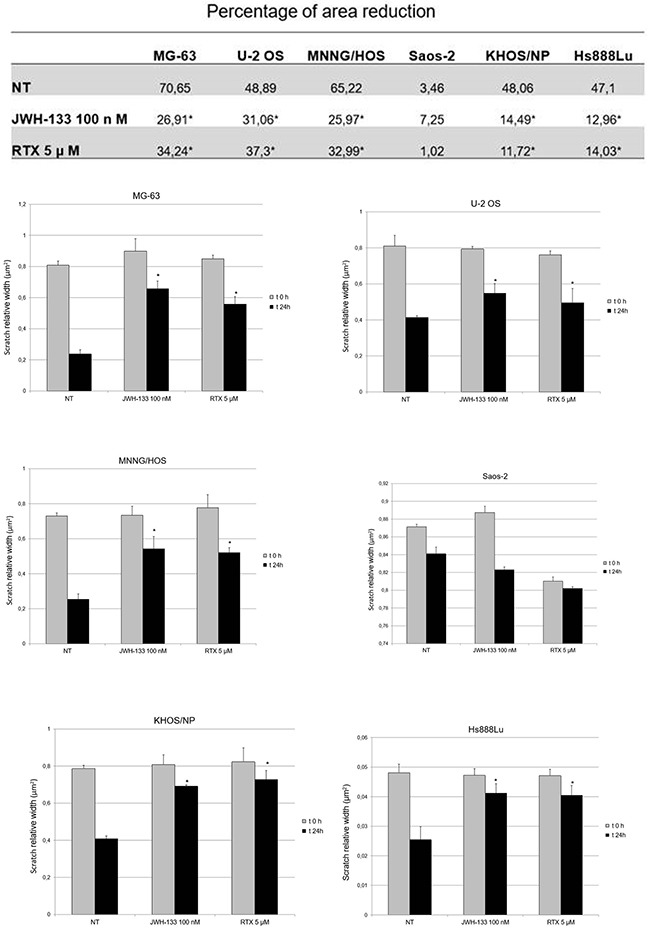
Effects of RTX and JWH-133 on Human OS cell lines migration capacity The Table shows the mean percentage of reduction of the scratched area, in the different OS cell lines after 24h treatment with JWH-133 [100nM], RTX [5μM] and in the Non-Treated cells, compared to T0 area width. The area (μm^2^) of the scratch was measured with Motic images plus 2.0 Software. The Histograms display the relative quantification of the scratch width (μm^2^) after 24h, compared with the initial width area. Data derived from three different assays. A t-test has been performed to evaluate the statistical differences among groups. * indicates p ≤ 0.05 compared to the untreated control (NT).

**Figure 7:**
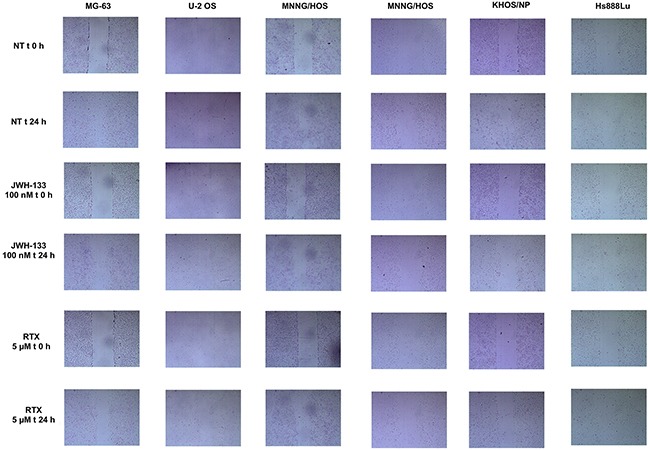
Scratch Test on Human OS cell lines at T0 and after 24h of JWH-133 and RTX treatments The representative images of scratch relative width (μm^2^) are displayed. Cells were plated in a 6-well plate. A single scratch was made at the confluence of 70%-80%. The scratch was monitored and photographed at 0 and 24 h post-treatment with JWH-133 [100nM] and RTX [5μM]. Images were taken on a AE2000 inverted microscope at 4x magnification.

OS cell lines were treated with EC/EV compounds and their migration capacity was measured after performing a scratch at the center of a 70% - 80% confluent well. We measured the migration capacity in terms of % of reduction of the scratched area (Figure 6). Hence, the lower is the percentage of area reduction, the lower is the migration observed in the well (Figure 7).

Compared with the untreated cells of the same line, we observed a reduction in migration capacity, with both treatments (RTX and JWH-133), in all cell lines except Saos-2. The reduction in cells migration was statistically significant (Figure 6).

#### mRNA levels of genes involved in invasion/migration capacity (Notch-1 and MMP-2)

**Figure 8:**
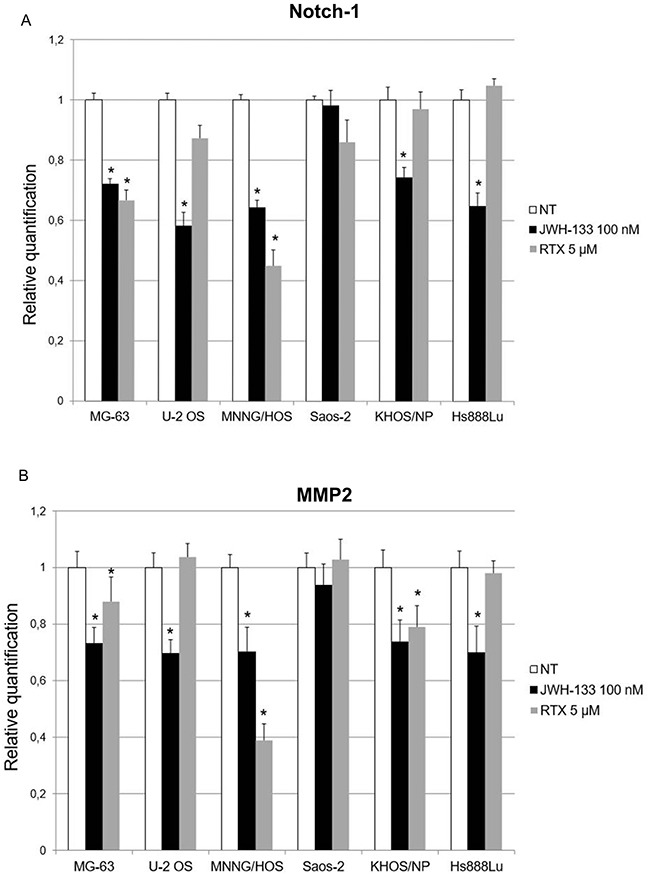
Effects of JWH-133 and RTX treatments, on Notch-1 and MMP-2 mRNA expression levels Notch-1 **(A)** and MMP-2 **(B)** mRNA expression levels in OS cell lines determined by Q-PCR after JWH-133 [100 nM] and RTX [5 μM] treatments. Results were normalized for the housekeeping gene β-actin and are showed as mean ± SD of three independent experiments. A t- test has been performed to evaluate the statistical differences in the levels of gene expression among groups. * indicates p ≤ 0.05 compared to the untreated control (NT).

To evaluate the possible role of EC/EV drugs on cell invasion and migration we analyzed the expression levels of Notch-1 and MMP-2, after RTX [5 μM] and JWH-133 [100 nM] treatments. The incubation with RTX in the first 24 hours of exposure reduced significantly the expression of Notch-1 in MNNG/HOS and MG-63, while treatment with JWH-133 down regulated this gene in all cell lines except Saos-2. Also MMP-2 showed a significant decrease in all cell lines studied except for Saos-2 after JWH-133 exposure. RTX treatment reduced MMP-2 expression in MNNG/HOS, KHOS/NP and MG-63 in a statistically significant manner (Figure 8).

## DISCUSSION

OS is the most common bone tumour in paediatric age, with high metastasis rate and poor life quality, due to chemotherapy-associated effects [1, 2]. For these reasons new therapeutic approaches are needed [3, 4, 6]. We analyzed the therapeutic potential of EC/EV drugs JWH-133 and RTX. JWH-133 is a potent CB2 selective agonist, while RTX is an analog of capsaicin, a vanilloid agonist. We tested these compounds in human OS cell lines. Tumour derived cell lines can be used as excellent *in vitro* models as long as they are representative of the original tumor [41]. Usually cell lines are questioned because of their additional genetic alterations (*in vitro* obtained). Mohseny *et al*. assayed a list of well genetically characterized cell lines, more representative of clinical OS, that could be used to establish valid *in vivo* and *in vitro* models. Among those, we used MG-63, U-2 OS, MNNG/HOS, Saos-2 [41]. Moreover, we used also a cell line easy to transfect, KHOS/NP, and a cell line derived from a OS lung metastasis, Hs888Lu [42]. This is the first study where two different compounds acting on the EC/EV system have been tested on six different OS cell lines and their effect has been evaluated via four different techniques. We showed, for the first time, that all six evaluated OS cell lines express EC/EV system and can be stimulated by EC/EV drugs like previously demonstrated in human MSCs and OBs cells [31, 40].

Cannabinoids exert a direct anti-proliferative effect on tumor of different origins [24]. Moreover, CB2 activation may be involved in THC-induced anti-inflammation in OS cell lines [15] and TRPV1 stimulation has already been shown to lead to cell death by activating Calcium channels, causing cell swelling and membrane lyses [43]. Apoptosis is mediated by multiple elements for instance Caspases are crucial mediators of programmed cell death [44]. We demonstrated an increase in Apoptosis and Caspase-3 expression by JWH-133 at a low dosage and/or by RTX in the different lines. Recent findings reveal that pAKT is enhanced in primary tumors and it is implicated in developing pulmonary metastasis [45]. Total Akt expression levels unchanged or increased after RTX and JWH-133 treatments, which is consistent with results obtained in other studies reported in literature [46–49], while interestingly, CB2 and TRPV1 receptor stimulation reduced pAKT protein expression in all of the cell lines studied, including the line derived from OS lung metastasis. Taken together, these data show an important switch toward the programmed cell death in all the OS cell lines studied.

Moreover, the most striking result in favor of administration of these compounds in OS is the cell migration inhibition demonstrated with the Scratch Test. Several studies have already demonstrated that cannabinoids are capable of inhibiting cell migration and metastasis in different types of cancer [25, 26]. RTX and JWH-133 demonstrated to be efficient in reducing the migration in all OS cell lines used for this study. In Saos-2 the result has no statistical significance, but this is most likely due to the fact that these cells duplicate very slowly (37 hours duplication time [50]) so probably in the future, the test can be repeated on these cells in a different time frame. The cannabinoid anti-tumor activity also acted through Notch-1 signalling pathway inactivation and MMP-2 down regulation. Notch signalling pathway is critical in cell proliferation and apoptosis [51, 52] and the MMPs are important in facilitating tumour invasion [53] so that active MMP-2 is considered a cancer metastasis indicator [54]. Previously, Niu*et al*. evaluated the effect of a synthetic, non selective, cannabinoid receptor agonist with psychotropic effects, WIN-55, 212-2, on these genes in a single OS cell line, MG-63 [35]. In prospective of a clinical application, instead, we choose two drugs selectively acting on TRPV1 and CB2 receptors and so probably without psychotropic effects. As previously mentioned, chemotherapy is known to reduce BMD and cause osteoporosis and bone fractures [7, 8]. Considering the important role of EC/EV system in bone metabolism [33, 34], we speculate that these drugs could reduce chemotherapy-related bone loss in a selective manner, significantly improving patient's life quality. Further in deep studies, combining the compounds used in this study with current conventional OS therapy, are required to investigate the precise molecular mechanism of these antitumor activities and whether this co-administration could be effectively applied in clinical practice. Moreover we found, consistently with others [21, 24, 55] that JWH-133 at low dosage reduces proliferation and induces apoptosis, while at higher dosage it has an opposite effect. So dosage will be a critical point when shifting to clinical practice. In conclusion, our results strongly support the potential of EC/EV system as new therapeutic target in OS, having demonstrated its capacity to interfere in tumor growth and invasion capacity by increasing/decreasing target genes involved in proliferation, migration and apoptosis.

## MATERIALS AND METHODS

### Cell lines

OS MG-63, U-2 OS, MNNG/HOS, Saos-2, KHOS/NP and Hs888Lu cell lines were purchased from Sigma Aldrich. The MG-63, KHOS/NP, MNNG/HOS cell lines were cultured in EMEM medium with 1 % Non Essential Amino Acids (NEAA), the Saos-2 and the U-2OS cell lines were cultured in McCoy's medium, the Hs888Lu cell line was cultured in DMEM medium. The complete culture medium containing 10% fetal bovine serum (FBS), supplemented with 100 U/ml penicillin (Gibco), 100 U/ml streptomycin (Gibco) and 2mM L-glutamine (Euroclone). Cells were culturedat 37°C in a humidified atmosphere with 5% CO_2_. After 48-hours adhesion cells were harvested using trypsin, washed and counted on a microscope using a Burker haemocytometer and 3,8 × 10^4^ cells per well, were plated in a 12 well cell culture multiwell. Once 80% confluence was reached, JWH-133 and RTX were added at the following concentrations: JWH-133 [100 nM, 1 μM and 5 μM], RTX [2.5 μM, 5 μM and 7 μM] and cells were harvested at 24h for mRNA isolation, protein extraction, and Muse® “Annexin V and Dead Cell Assay Kit” (Millipore).

### Drugs and treatments

Resiniferatoxin (RTX- Potent analog of capsaicin, that is an agonist at vanilloid receptors) and JWH-133 (Potent CB2 selective agonist. Approx. 200-fold selective over CB1 receptors) were purchased from Tocris Bioscience (Bristol, UK). The powder was dissolved in dimethyl sulfoxide (DMSO) at a concentration of 50mM for JWH-133. RTX was dissolved in DMSO to a stock solution of 100mM. DMSO final concentration on cell cultures was 0.01%. Stock solutions were aliquoted and kept at −80°C for long-term storage. OS cell lines were treated with RTX [2.5 μM, 5 μM and 7 μM] and JWH-133 [100 nM, 1 μM and 5 μM]. For real time and western blotting studies EC/EV compounds were added alone at concentrations of RTX [5 μM] and JWH-133 [100 nM]. We choose only these treatments considering the results obtained in the apoptosis assay. Non-treated cultured cell lines were maintained in incubation media during the relative treatment time with and without vehicle (DMSO 0.01%).

### Annexin, count and viability

Apoptosis has been evaluated by a fluorometric assay on the Muse cell analyser machine with the “Cell dead and Annexin V Assay Kit”. Test was performed after 24 h of EC/EV compounds exposure. EC/EV active compounds were added alone at the following concentrations: JWH-133 [100 nM, 1 μM and 5 μM], RTX [2.5 μM, 5 μM and 7 μM]. The Muse™ Annexin V & Dead Cell Assay utilizes Annexin V to detect phosphatidylserine (PS) on the external membrane of apoptotic cells. A dead cell marker, is also used as an indicator of cell membrane structural integrity, 7-amino-actinomycin D (7-AAD). Briefly, 100μL of a cell suspension (1 × 104 cells/mL) was mixed with100 μL of Muse™ Annexin V & Dead Cell Reagent and incubated for 20 minutes at room temperature in dark. The results, automatically displayed, were analyzed with “Muse 1.4 Analysis” software for data acquisition and analysis.

### Total RNA extraction and reverse transcription quantitative polymerase chain reaction (RTqPCR)

Following treatment with JWH-133 [100 nM] and RTX [5 μM] and incubation for 24 h at 37°C with 5% CO2, OS cells were harvested. Cells without treatment served as the control group. The total RNA was extracted using Quiazol® (Quiagen) following the manufacturer's instructions. EasyScript™ cDNA Synthesis Kit (abm) was used to synthesize from approximately 1000ng mRNA, the first strand cDNA. The transcript levels of Notch-1, matrix metalloproteinase-2 (MMP-2) and Caspase-3 (CASP3) were detected by RT-qPCR using a CFX96 Real-Time PCR system (Bio-Rad) using I-Taq Universal SYBR® Green Master Mix (Bio-Rad). The cycling conditions were 10 min at 95°C (initial denaturation) followed by 40 cycles of 15 sec at 94°C (denaturation) and 1 min at 68°C (annealing/extension/data collection). The β-Actin gene served as the reference gene for the normalization of the real-time PCR products. The PCR primers used to detect each gene were designed using Primer 3 program and synthesized by Sigma Aldrich (CB2_F 5’-AAGGCTGTCTTCCTGCTGAA-3’, CB2_R 5’-CACAGAGGCTGTGAAGGTCA-3, TRPV1_F 5’-C TGCAGAAGAGCAAGAAGCA-3’, TRPV1_R 5’-ATG GCTTTCAGCAGACAGGT-3’, Caspase3_F

5’-TTGTGGAATTGATGCGTGAT-3’, Caspase 3_R 5’-TGGCTCAGAAGCACACAAAC-3’, Notch-1_F 5’-TC CTTCTACTGCGAGTGTCC-3’, Notch-1_R 5’-TCGTT ACAGGGGTTGCTGAT-3’, MMP2_F 5’-GACCGCG ACAAGAAGTATGG-3’, MMP2_R 5’-GTTGCCCAGG AAAGTGAAGG-3’, β-Actin_F 5’-GCGAGAAGATG ACCCAGATC-3’, β-Actin_R 5’-GGATAGCACAGC CTGGATAG-3’). The linearity and efficiency of the assays were tested over dilutions of input cDNA spanning five orders of magnitude. Assays were performed in triplicate. The dissociation curve analysis of amplification products was performed at the end of each PCR reaction to confirm the specificity of the amplification. The 2^-ΔΔCt^ method was used to analyze the data and obtain the relative gene expression levels compared to the controls.

### Scratch assay

Osteosarcoma MG-63, U-2 OS, MNNG/HOS, Saos-2, KHOS/NP and Hs888Lu cell lines were seeded in a six well plate (500.000 per well). Incubated 24 hours to let them attach in monolayer in the wells. A single scratch was made in each well with a 200 μl sterile pipette tip. The well has been washed with PBS and fresh media containing RTX [5 μM] and JWH [100nM] has been added. Fresh medium without any drug was used as control (Labelled as NT: Non-Treated). Images were taken 24 hours later with an AE2000 microscope (Motic) and analyzed with Motic Images plus 2.0 Software.

### Western blotting

Proteins were extracted from treated and non-treated OS cell lines using RIPA Lysis Buffer (Millipore) and following the manufacturer's instructions. Akt, pAKT and Caspase-3 proteins were characterized in total lysates from cell line cultures by Western blotting. Membranes were incubated overnight at 4°C with rabbit polyclonal anti pAKT antibody (1:1000 dilution; Cell Signaling), rabbit policlonal anti Akt antibody (1:200 dilution; Santa Cruz) and rabbit anti Caspase-3 antibody (1:200 dilution; Cell Signaling). Reactive bands were detected by chemiluminescence (Immobilon western Millipore) on a C-DiGit ® Blot Scanner (LI-COR Biosciences). A mouse polyclonal anti β-Tubulin antibody (1:1000 dilution; Sigma) was used to check for comparable protein loading and as a housekeeping protein. Images were captured, stored, and analyzed using “Image studio Digits ver. 5.0” software.

### Statistical analysis

Results are expressed as means ± S.D. The experiments were run in triplicate. Statistical analyses were performed using Student's *t* test to evaluate differences between quantitative variables. A *p* value less than 0.05 (*) or 0,01 (**) were considered statistically significant.

## SUPPLEMENTARY MATERIALS FIGURE


