# Supplementary material for: Anti-proliferative, pro-apoptotic and anti-invasive effect of EC/EV system in human osteosarcoma
Source: Oncotarget. 2017 Apr 13;8(33):54459–71. doi: 10.18632/oncotarget.17089 (PMC5589594; doi:10.18632/oncotarget.17089)
Supplement: Supplementary file 1 [file oncotarget-08-54459-s001.pdf]

Anti-proliferative, pro-apoptotic and anti-invasive effect of EC/EV system in human osteosarcoma

SUPPLEMENTARY FIGURE

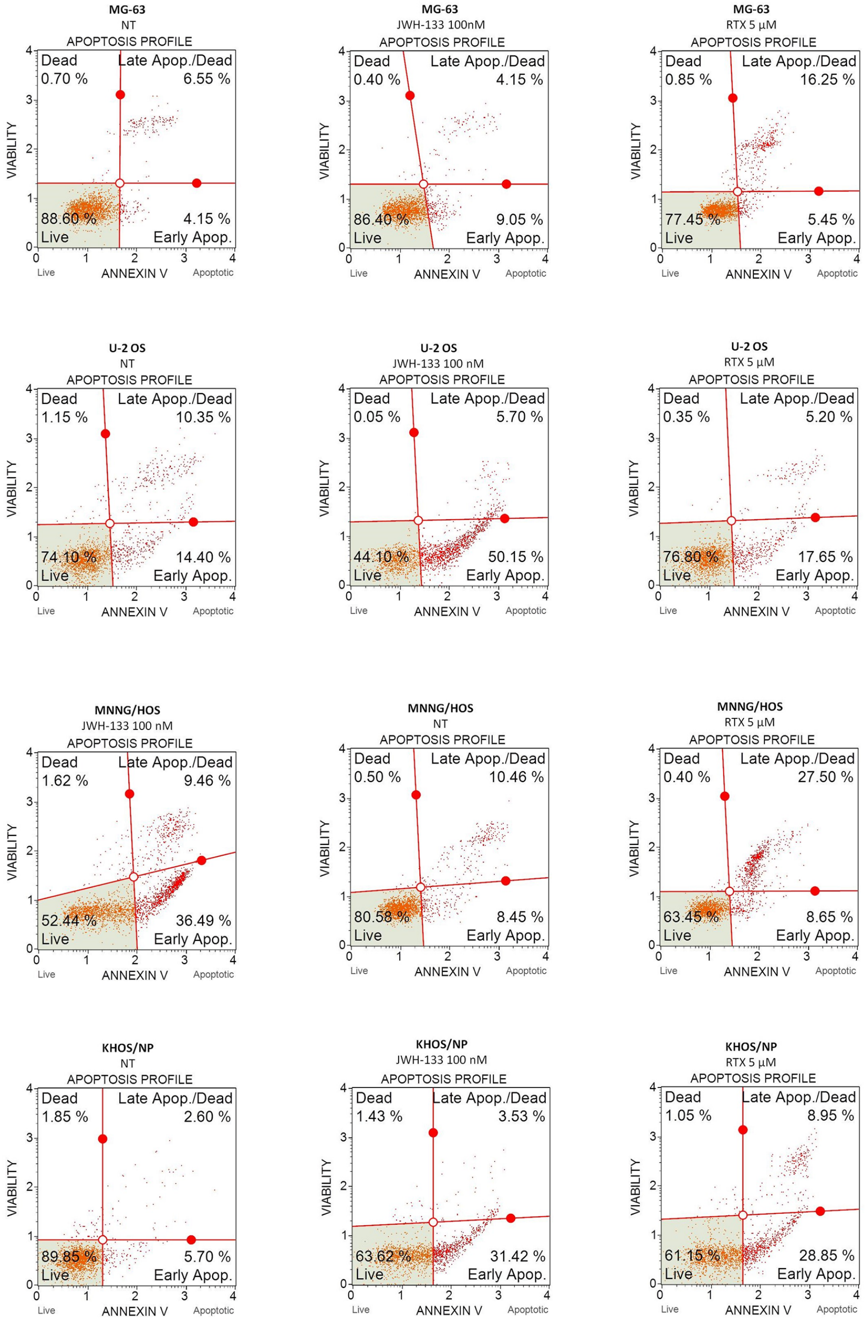

Supplementary Figure 1: Scatter Plots of Apoptosis Assay in JWH-133 and RTX treated OS cell lines. The panels show cytofluorimetric scatter plots representing percentages of apoptosis for MG-63, U-2 OS, MNNG/HOS, Saos-2 and KHOS/NP cell lines. Apoptosis assay was performed using "Cell dead and Annexin V Assay Kit" (Millipore) following the manufacturer's protocol. The results were obtained with "Muse Count & Viability" software.
